# Supplementary material for: Progressive chromosome shape changes during cell divisions
Source: EMBO Rep. 2025 Sep 23;26(21):5199–215. doi: 10.1038/s44319-025-00577-4 (PMC12592473; doi:10.1038/s44319-025-00577-4)
Supplement: Supplementary file 2 — Expanded View Figures [file 44319_2025_577_MOESM2_ESM.pdf]

Expanded View Figures

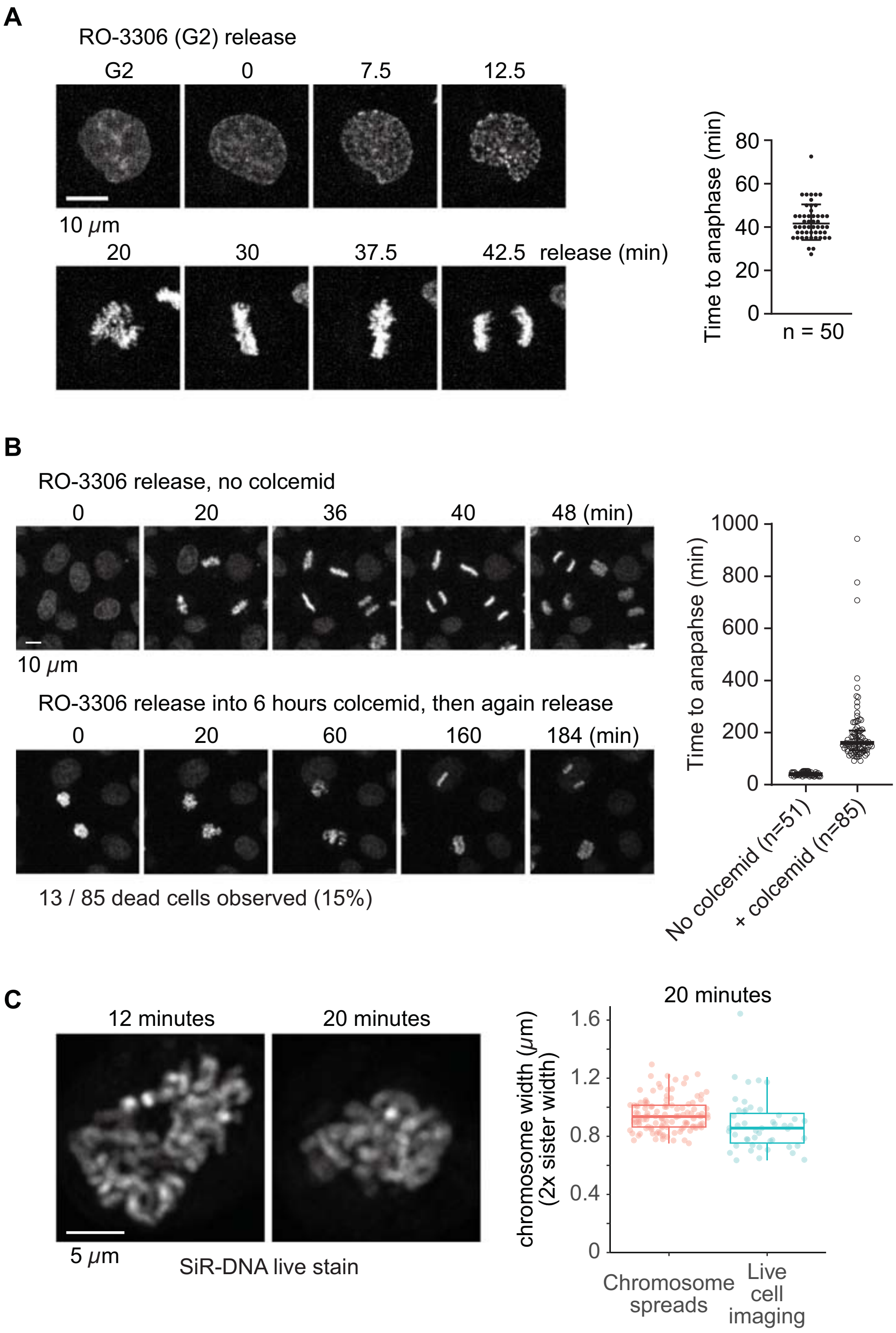

Figure EV1. Additional chromosome analyses during mitotic progression.

(**A**) Timing of chromosome segregation. Cells were synchronized in G2 by RO-3306 treatment and released as in the experiment shown in Fig. 1, but in the absence of colcemid. A time series of images of a cell traversing through mitosis is shown. The time of anaphase onset was determined in 50 cells. Each measurement result is shown. The center line indicates the mean, the error bar the standard deviation. (**B**) Cells remain viable and complete cell division after release from 360 min colcemid arrest. Example time series of fields of cells are shown, either released from G2 or released following an additional 360-minute colcemid block, and the time from release to anaphase onset was recorded in the indicated numbers of cells. Each measurement result is shown. The center lines indicate the means, the error bars the standard deviations. (**C**) Chromosomes visualized in live cells using the SiR-DNA stain, following release from G2 synchronization by RO-3306. Example images are shown at 12 and 20 min after release. Chromosome widths measured at 20 min after release are compared to those measured on fixed and spread DAPI-stained chromosomes (compare Figs. 1 and 2B). The box plots show the medians (center), interquartile ranges (bounds of boxes) and 90th percentile ranges (whiskers). The slightly wider appearance of chromosomes after spreading could arise not from distortion, but if chromosomes whose diameter is not perfectly round oriented themselves with their flat side on the surface.

 © The Author(s)

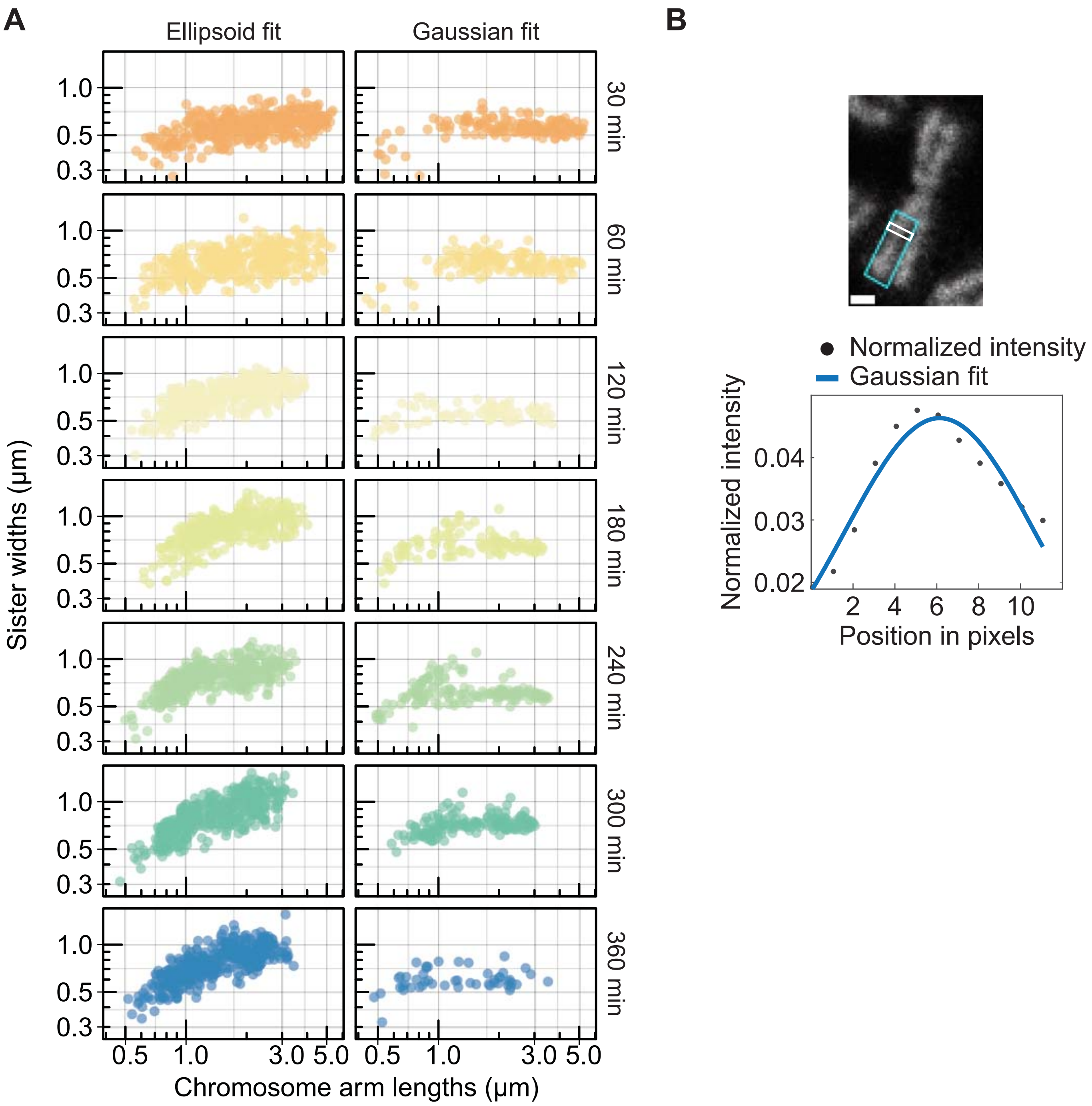

Figure EV2. Comparison of chromosome width measurements by ellipsoid and moving Gaussian fitting.

(**A**) Ellipsoid fit measurements reported in Fig. 2 are compared to measurements obtained by a sliding Gaussian fit of a subset of the same chromosome arms. Both methods reveal gradual chromosome shortening and widening over time, and that longer arms become progressively wider than shorter arms. The Gaussian fits suggest that the width plateaus with respect to length, for the longest arms. A plateau width of the longest arms is compatible with the idea that these arms widen at a uniform rate and are still on course to approach, but have not yet reached, their final steady state width. However, the incomplete sampling makes it difficult to quantitively compare the two sets of measurements, thereby preventing a firm conclusion. (**B**) A limitation of the sliding Gaussian approach is the fact that DAPI intensity from the neighboring sister chromatid often distorts the fit (see the example—scale bar, 1 µm). For this reason, and because other chromosome arms had to be entirely excluded from the sliding Gaussian fit approach if they were bent, we use the ellipsoid fit measurements that could be applied to all chromosome arms for subsequent analyses.

© The Author(s)

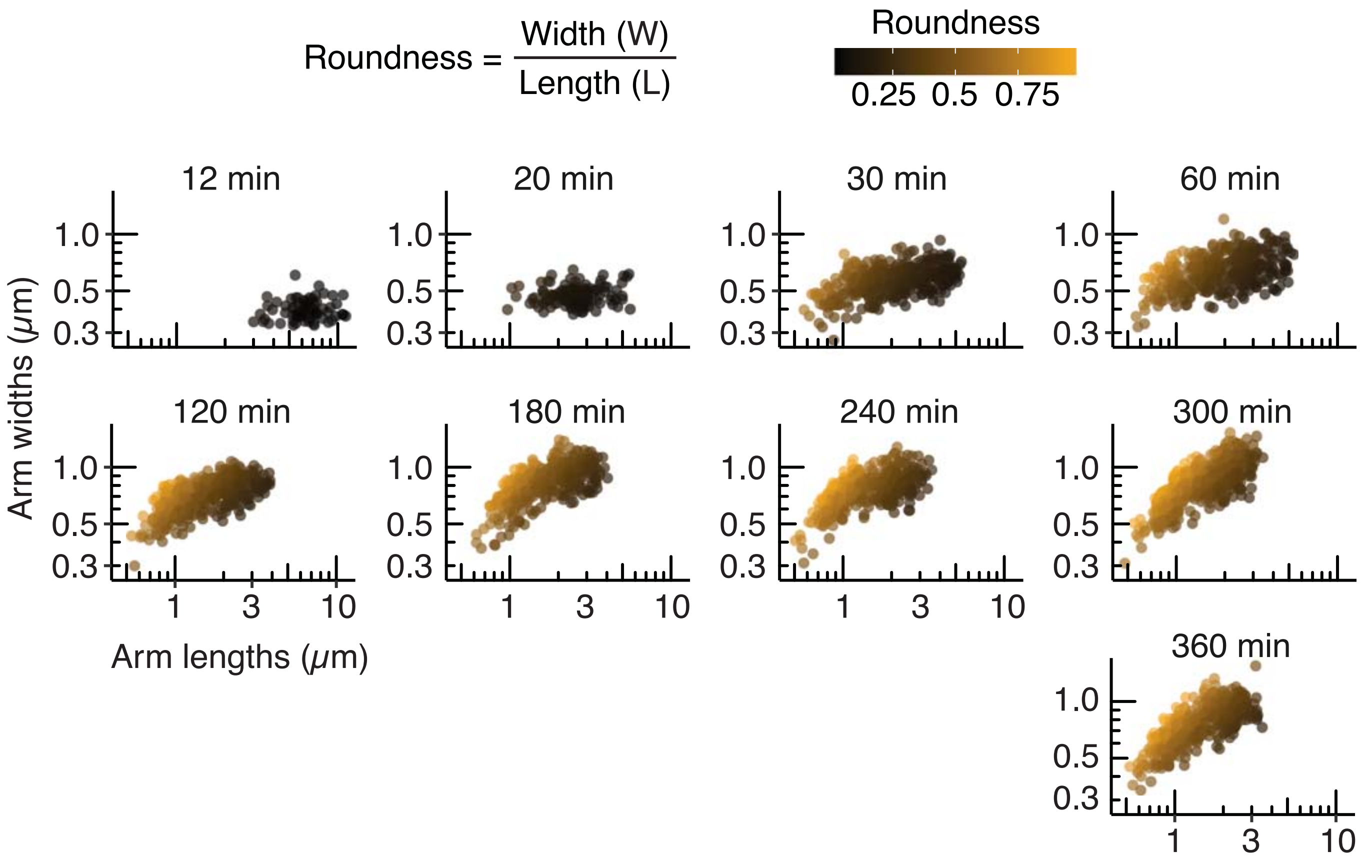

Figure EV3. Chromosome roundness over time.

Chromosome width as a function of length, at the designated times are shown, with increasing roundness indicated as hues of black to yellow.

 © The Author(s)

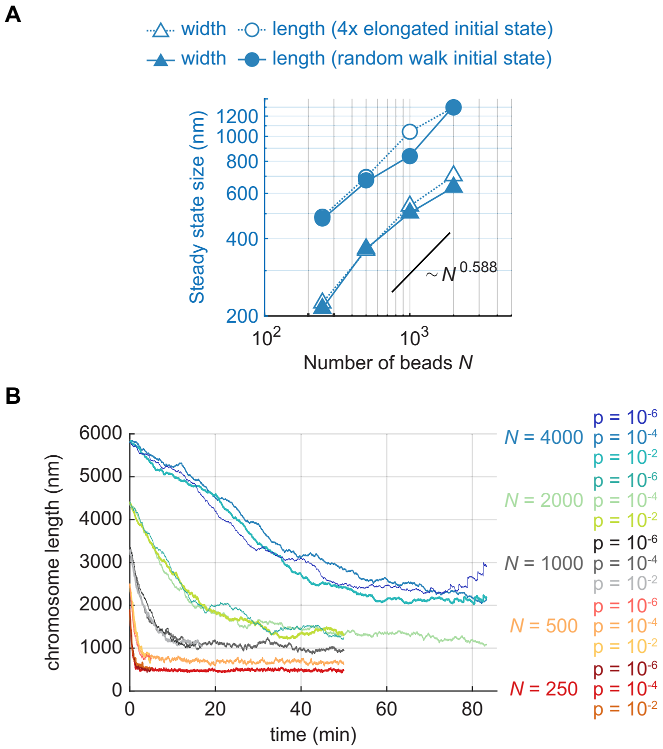

Figure EV4. Comparison of different initial states, and looping probabilities, on the chromatin simulations.

(**A**) Steady state chromosome lengths and widths, when simulations of increasing chain lengths were started from either an unstretched random conformation, or from a four times elongated conformation. The theoretically expected scaling behavior of random self-avoiding polymers is indicated for comparison. (**B**) The time change of simulated polymer dimensions is robust to changes in condensin's loop capture probability. As Fig. 6A, but loop capture probabilities two magnitudes higher or lower than the standard probability were simulated.
